# Participation of Intussusceptive Angiogenesis in the Morphogenesis of Lobular Capillary Hemangioma

**DOI:** 10.1038/s41598-020-61921-3

**Published:** 2020-03-19

**Authors:** Lucio Díaz-Flores, Ricardo Gutiérrez, Miriam González-Gómez, M. A. Pino García, José Luis Carrasco, Lucio Díaz-Flores, Juan F. Madrid, Hugo Álvarez-Argüelles

**Affiliations:** 1 Department of Basic Medical Sciences, Faculty of Medicine, University of La Laguna, Tenerife, Spain; 2 Department of Pathology, Eurofins® Megalab–Hospiten Hospitals, Tenerife, Spain; 3 Department of Cell Biology and Histology, School of Medicine, Regional Campus of International Excellence. “Campus Mare Nostrum”, University of Murcia, Espinardo, Spain

**Keywords:** Mechanisms of disease, Peripheral vascular disease

## Abstract

In lobular capillary hemangioma (LCH), misnamed pyogenic granuloma, only sprouting angiogenesis (SA) has been considered. We assess the occurrence of intussusceptive angiogenesis (IA) in LCH and whether IA determines the specific and other focal patterns in the lesion. For this purpose, we study specimens of 120 cases of LCH, using semithin sections (in 10), immunohistochemistry, and confocal microscopy (in 20). In addition to SA, the results in LCH showed (1) intussusceptive phenomena, including pillars/folds and associated vessel loops, which encircled interstitial tissue structures (ITSs). (2) Two types of evolved loops depending on interendothelial contacts from opposite walls: (a) numerous interendothelial contacts, alternating with capillary-sized lumens (main capillary pattern of the lesion) and (b) few interendothelial contacts, wide open lumens, and intravascular transport of pillars/folds, which were arranged linearly, forming septa (focal sinusoidal-like pattern) or were irregularly grouped (focal intravascular papillary endothelial hyperplasia, IPEH-like pattern). In conclusion, we demonstrate that IA participates in synergistic interaction with SA in LCH development and that the prevalence of specific intussusceptive phenomena determines the predominant capillary pattern and associated sinusoidal hemangioma-like and IPEH-like patterns in the lesion, which suggest a role of IA as conditioner of vessel tumour/pseudo-tumour morphology.

## Introduction

The two principal and complementary forms of angiogenesis in physiological and pathological conditions are sprouting angiogenesis (SA) and intussusceptive angiogenesis (IA). In addition to microvascular growth, IA participates in vascular morphogenesis and remodelling, including vessel arborization, branching remodelling and vessel segmentation^1–11^. Hallmarks of IA are intravascular tissue pillars, which split or remodel pre-existing or newly formed vessels. Two types of pillars have been established according to diameter: small (diameter ≤ 2.5 µm) and large (diameter > 2.5 µm) pillars^10,11^. Folds, which form pillars when spanning, are also intussusceptive phenomena. Different structures have been observed in association with pillar/fold formation, including (a) endothelial contacts, symmetric (kissing contacts) or asymmetric (peg-like contacts), established between endothelial cells (ECs) of opposite vessel walls; (b) meso-like intraluminal folds; (c) merged adjacent capillaries, with modified contacting walls; and (d) vessel loops, composed of a double-sheet layer of ECs, with virtual or different sized lumens, encircling interstitial tissue structures (ITSs)^3,6,10,12,13^. Likewise, secondary structures may form from pillars and folds, as occurs with intravascular meshworks of processes, septa and pillar aggregates^5,14–16^.

SA and IA are complementary mechanisms, with synergistic interaction^15,17^. In the chick chorioallantoic membrane, developmental avian kidney, lung and zebrafish caudal vein plexus, and in the rat femoral vein after PGE2 and glycerol perivenous administration, IA was shown to participate in capillary expansion following an SA phase^2,13,15,18,19^. Unlike SA, the participation of IA has generally been underestimated in vascular growth in vessel pathology, principally in vessel tumours and pseudotumours. Recently, we extended the exploration of IA to several human vessel diseases, including intravascular papillary endothelial hyperplasia (IPEH)^14^, dilated hemorrhoidal veins in hemorrhoidal disease^5^ and sinusoidal hemangioma^16^. Pillars and ITSs (termed by pathologists as papillae) and several associated structures were seen in these processes. In an experimental model resembling IPEH, we studied the sequential evolution of the pillar/fold/papilla formation, observing that SA phenomena occur in an initial phase, followed by IA^15^. In addition, sinusoidal hemangioma and IPEH shared morphologic findings and similar histogenesis, combining sprouting and intussusceptive angiogenesis^16^. We also proposed that the pattern of the processes outlined above is determined by the prevalence of certain intussusceptive findings. Thus, the morphologic pattern of IPEH and sinusoidal hemangioma depends on the expression and arrangement of pillars and associated structures: pillars (papillae) irregularly grouped in IPEH and incomplete intravascular septa formed by linearly grouped pillars in sinusoidal hemangioma^14,16^.

Lobular capillary proliferations are observed in some vascular tumours/pseudotumours. An example is the lobular capillary hemangioma (LCH), misnamed pyogenic granuloma. LCH grows rapidly, presents mitotic activity and can regress spontaneously. SA has been demonstrated in this process, whereas IA has not been considered. A study to assess whether IA occurs in LCH with such a different lobular capillary pattern from IPEH and sinusoidal hemangioma is not only of interest to explain LCH histogenesis, but also to contribute to the concept that variations of a common intussusceptive mechanism can condition the morphologic expression of vessel tumours/pseudotumours. To reinforce this hypothesis, this study should be expanded to investigate the presence of zones with other histologic patterns in LCH.

Given the above, the objectives of this study are (a) to assess whether IA participates in LCH and whether there is synergistic interaction with SA, (b) to explore the coexistence of other histologic patterns in LCH lesions, and c) to establish whether the prevalence of certain intussusceptive findings determines the morphological pattern(s) in LCH.

## Results

### General characteristics of LCH

**Figure 1:**
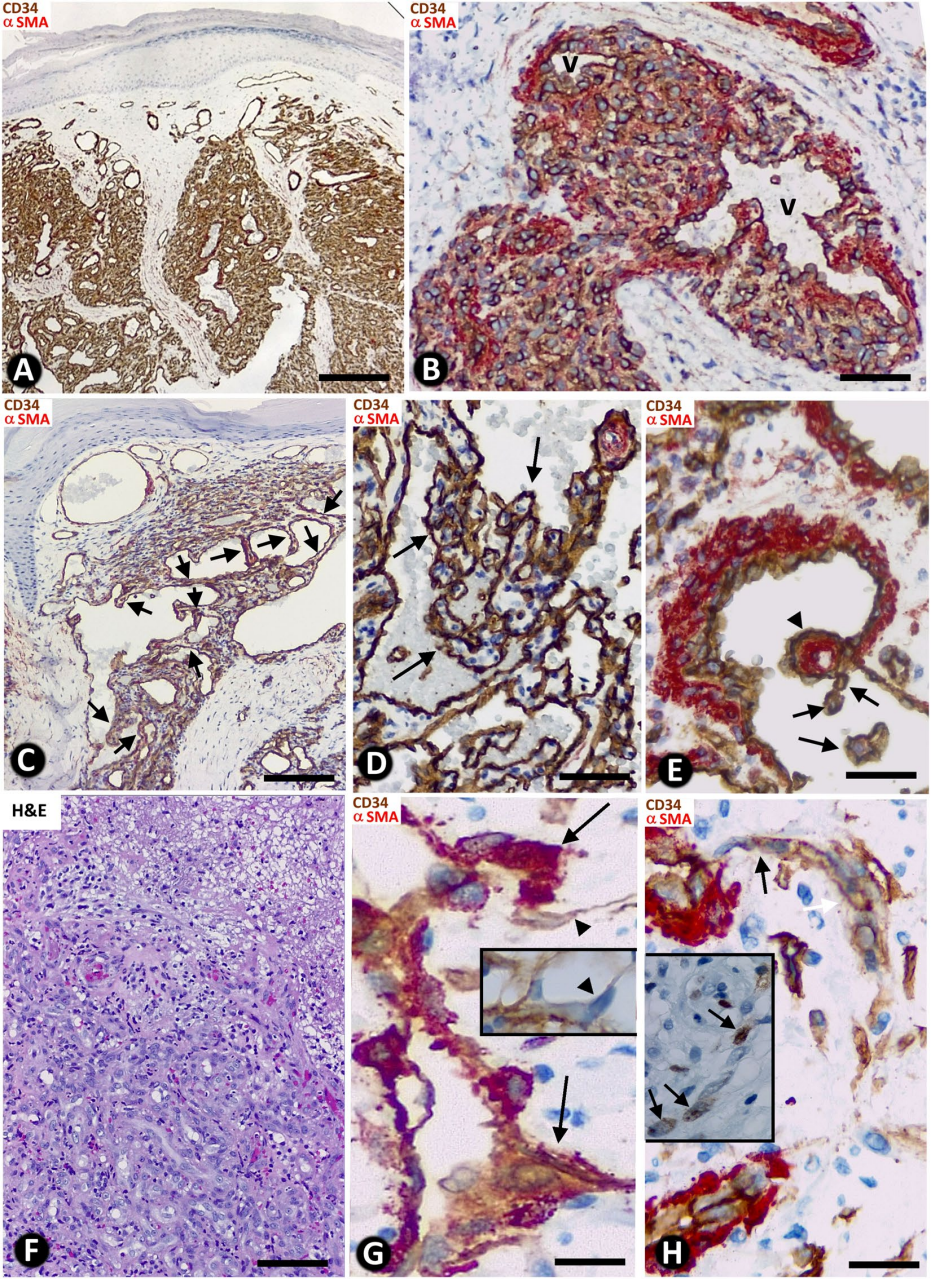
General characteristics of lobular capillary hemangioma (LCH). (**A**) Several lobules are observed in the lesion (lobular pattern). (**B**) In a lobule, presence of a branched venule (v) and numerous capillary-sized vessels, showing ECs (brown) and pericytes (red). (**C**) A zone with sinusoidal hemangioma-like morphology is observed, presenting wide vascular spaces with pillars, some of which are arranged in a linear fashion, forming incomplete septa (arrows). (**D**) An IPEH-like zone with numerous pillars irregularly arranged(arrows) in a wide vascular space. (**E**) CD34^+^ ECs (brown) and αSMA^+^ smooth muscle cells (red) are observed in the vessel walls. A large (arrowhead) and small pillars (arrows) are present. (**F**) Zone of ulceration with capillary-sized vessels, fibrin deposits and inflammatory infiltrate. (**G**) Sprouts are observed from a mother vessel (outward growth - arrows). In one of them, note a tip EC growing toward the interstitium (arrowhead). In the insert, the collagen IV stained basal membrane disappears in a tip EC (arrowhead). (**H**) Stalk ECs from a mother vessel are observed initiating a loop (arrows). In the insert, stalk ECs expressing ki67 in a loop are observed (arrows). (**A** to **E**,**G**,**H**) Double-staining with anti-CD34 (brown) and anti-αSMA (red). (**F**) H&E staining. Bar: (**A**,**C**) 160 µm; (**B**,**F**) 25 µm; (**D**,**E**,**G**,**H**) 20 µm.

**Figure 2:**
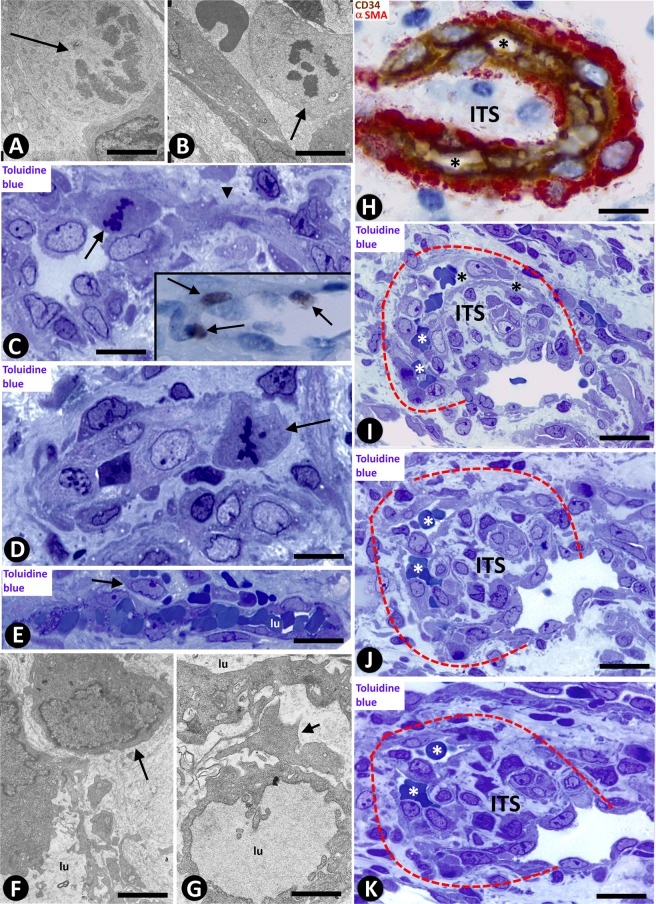
(**A**–**D**) Mitoses are observed in a stalk cell of a loop (A, arrow), and in endothelial cells (B and C, arrows) and in a pericyte (D, arrow) of mother vessels. In the insert of C, note cells expressing ki67 (arrows) in the wall of a mother vessel. In C, note a sprout emerging from a mother vessel (arrowhead). (**E**–**G**) Incorporation of pericytes or their processes (arrows) around ECs and formation of new vessel lumens (lu). (**H**–**K**) Vessel loops surrounding interstitial tissue structures (ITSs) are observed in double-staining (**H**) and in serial semi-thin sections (a dashed line delimits the loop, (**I**–**K**). In H, the loop encircling an ITS shows CD34^+^ ECs (brown) and αSMA^+^ pericytes (red). Perforated and unperforated interendothelial contacts from the opposite walls of the loops are seen alternating with zones in which the loop lumen (asterisk) is open (**H**–**K**). Note the presence of red blood cells in the open spaces of the loop (**I**–**K**). (**A**,**B**,**F**,**G**) Ultrathin sections. Uranyl acetate & Lead citrate. (**C**–**E**,**I**–**K**) Semithin sections stained with Toluidine Blue (**I**–**K**) serial sections). (**H**) Double-stained section with anti-CD34 (brown) and anti-αSMA (red). Bar: (**A**,**B**,**F**,**G**) 4 µm; (**C**,**D**) 10 µm; (**H**) 20 µm; (**E**–**K**) 25 µm.

Neovascularization in all cases of LCH (n: 120) adopted a predominantly lobular pattern (Fig. 1A,B). Zones with sinusoidal hemangioma-like (Fig. 1C) and IPEH-like (Fig. 1D) morphology were also seen. These zones were more evident in 20 cases of which 11 showed a sinusoidal hemangioma-like aspect and 9 an IPEH-like aspect. Occasional, small foci (low presence) of these associated patterns were seen in 32 cases (in 18 sinusoidal hemangioma-like and in 14 IPEH-like). In the remaining cases, no associated patterns were observed (see Table 1). In the predominant LCH pattern, each lobule showed numerous capillary-sized vessels and one variably branched venule (Fig. 1B), generally located in the centre. These vessels were lined by ECs expressing CD34 (Fig. 1B,E). Anti-αSMA^+^ mural cells (vascular smooth muscle cells and pericytes) were observed in the venules (Fig. 1E) and in capillaries (Fig. 1B). The lobules were separated by fibrous septa (Fig. 1A). A mixed inflammatory infiltrate and fibrin deposits were seen, predominantly in cases with ulceration (Fig. 1F). SA events were present, including outward growth from the vessel mother (Fig. 1G), with presence of tip endothelial cells (ECs) sprouting toward the interstitium (Fig. 1G), basal lamina degradation (insert of Fig. 1G), formation of a bilayer of stalk ECs (Fig. 1H), presence of mitoses in the stalk ECs (Fig. 2A) and in the ECs of the mother vessels (Figs. 2B,C) and in pericytes (Fig. 2D), incorporation of pericytes around the stalk ECs and formation of a new vessel lumen (Fig. 2E–G) with presence of red blood cells in their lumens (Fig. 2E). In addition, the proliferation index (ki67) was moderate/high in some loops (stalk cells) (insert Fig. 1H) and mother vessels (insert Fig. 2C).

**Table 1:** Patterns in LCH (LCH, sinusoidal hemangioma like and IPEH-like patterns) and characteristics of the principal structures typical for IA in the lesion, conditioning its morphology (Morphogenic role of IA).

MORPHOLOGIC PATTERNS		Types		LCH	SINUSOIDAL HEMANGIOMA-LIKE	IPEH
		Incidence		100%	24.16%	20.83%
		Extension		Extensive	Focal	Focal
**PRINCIPAL STRUCTURES TYPICAL FOR IA IN THE LESION**	**LOOPS**	Number		+++	++	++
		Lumen	narrow	+++	+	+
			wide	+	+++	+++
		Endothelial contacts		+++	+/−	+/−
		Perforation of the endothelial contacts		+++	+/−	+/−
	**PILLARS**	Number		++	++	+++ (Myriad)
		Predominant size		Small and thin	Large	Giant, large, and thin
		Arrangement		Loop path	Linear, forming septa	Irregular, forming groups
		Type and lumen size of vessels in which pillars are predominantly located		Loop Narrow lumen	Mother vessel Wide lumen	Mother vessel Wide lumen
	**ITSs**	Substrate	Interstitial tissue	+++	+	+
			Vessel wall components	+/−	+++	+++
			Fibrin	+/−	+	+++
		Location	Extravascular (non-transported into the vessel lumen)	+++	+/−	+/−
			Intravascular (transported into the vessel lumen)	+/−	+++	+++

**Figure 3:**
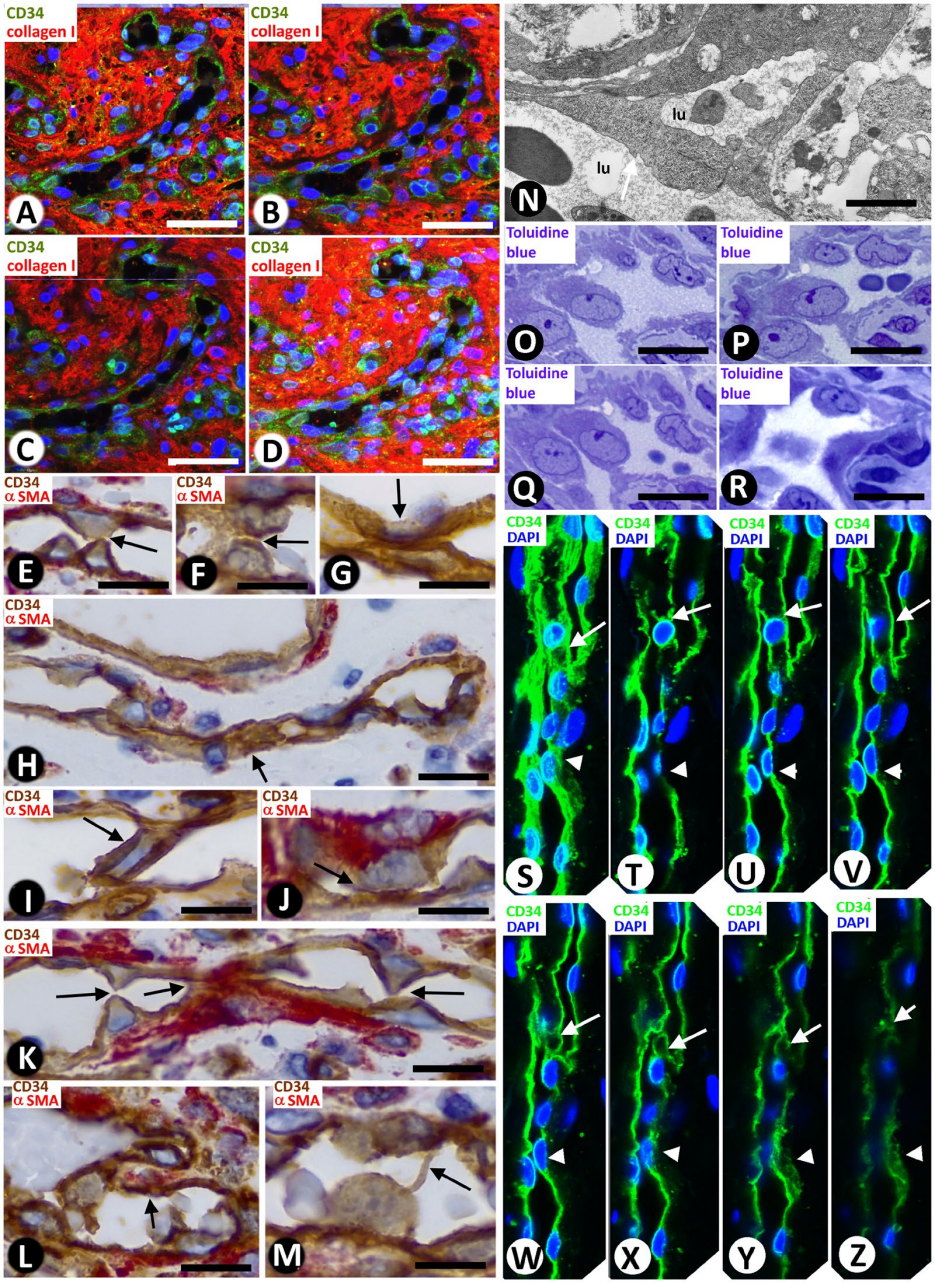
(**A**–**D**) Images showing the spatial path of a loop zone, in single (**A**–**C**) and whole-mount (**D**) views in confocal microscopy (frontal view, 6 µm section, immunofluorescence label with anti-CD34, green, and anti-collagen I, red, DAPI: blue). (**E**–**M**) Contacts between ECs from opposite walls of the loops (arrows). Note that the contacting ECs may have triangular (**E**), ovoid (**F**) or flattened (**G**) morphology in the histological sections and that planar contact may be extensive (**H**). Contacts can be symmetric (**E**) or asymmetric (**I**,**J**), in proximity (**K**) or not, and can acquire a peg-like aspect when ECs surround connective tissue (**L**). (**M**) Occasionally, smaller transcapillary cytoplasmic projections (antenna-like or filipodia-like) were seen. (**N**) Ultrastructural image of an endothelial projection (arrow) separating two luminal spaces (lu) of a loop. (**O**–**R**) Serial semithin sections showing an EC projecting intraluminally (in Figs. 4A,B, in which a perforated contact is shown, the region in O-R also appears in the image with a portion of the projecting cell -Fig. 4A- and with no presence of this projecting cell -Fig. 4B-). (**S**–**Z**) Single (**T**–**Z**) and whole-mounted (**S**) views in confocal microscopy (frontal view, 10 µm section and CD34 staining, DAPI: blue) showing the appearance and disappearance of endothelial contacts (arrowhead), including a peg-like structure (arrow). (**E**–**M**) Double-staining with anti-CD34 (brown) and anti-αSMA (red). (**N**) Ultrathin section. Uranyl acetate and Lead citrate. (**O**–**R**) Semithin sections stained with Toluidine Blue. Bar: A-D, 30 µm; (**E**–**M**,O–**Z**) 10 µm; (**N**) 8 µm.

**Figure 4:**
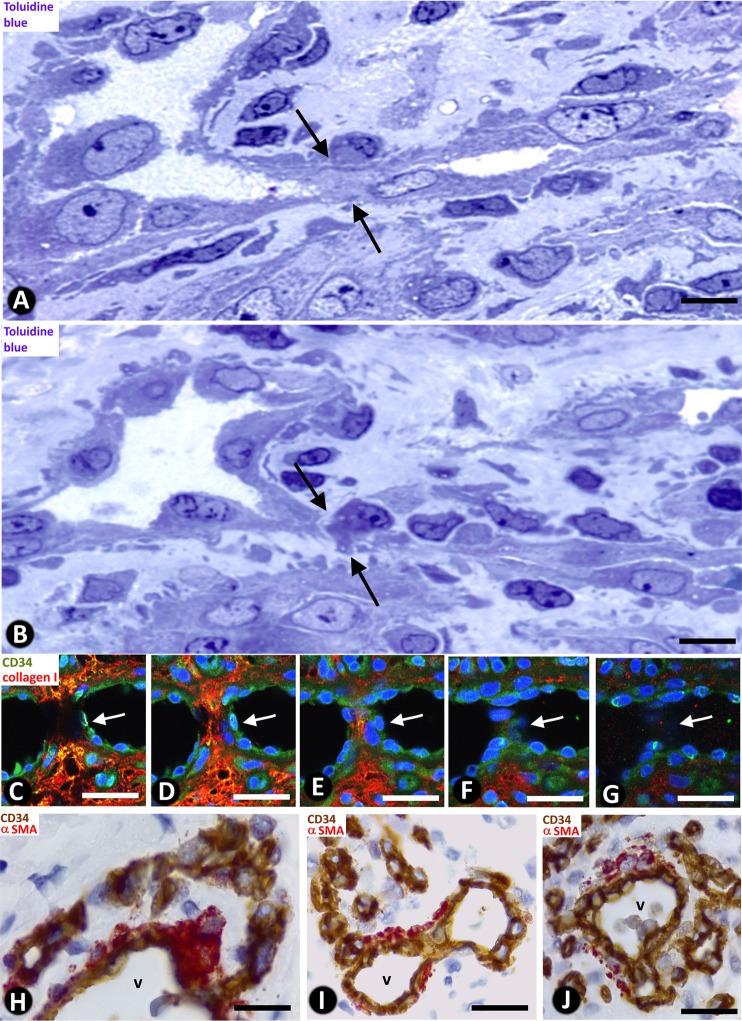
Perforations of interendothelial contacts and loop fragmentation into capillary-sized vessels. (**A**,**B**) An interendothelial contact (A, arrows) and its perforation (B, arrows) are shown in two sections obtained from serial semithin sections. Note the presence of neighbouring interstitial cells in the perforated zone. (**C**–**G**) Images in confocal microscopy with double immunofluorescent label (CD34 and collagen I, DAPI: blue) demonstrating the appearance and disappearance of a pillar (arrows), with an endothelial cover (green) and a core with collagen I (red). (**H**–**J**) Images showing loop fragmentation in several capillary-sized vessels after perforation of interendothelial contacts. Note that the loops arise from venules (v) and that the capillary-sized vessels are arranged in a linear arciform path reminiscent of that of loops. (**A,B**) Semithin section stained with Toluidine Blue. (**C**–**G**) Immunofluorescent label with CD34, collagen I and DAPI. (**H**–**J**) Sections double-stained with anti-CD34 (brown) and anti-αSMA (red). Bar: (**A**,**B**) 8 µm; (**C**–**G**) 30 µm; (**H**–**J**) 35 µm.

**Figure 5:**
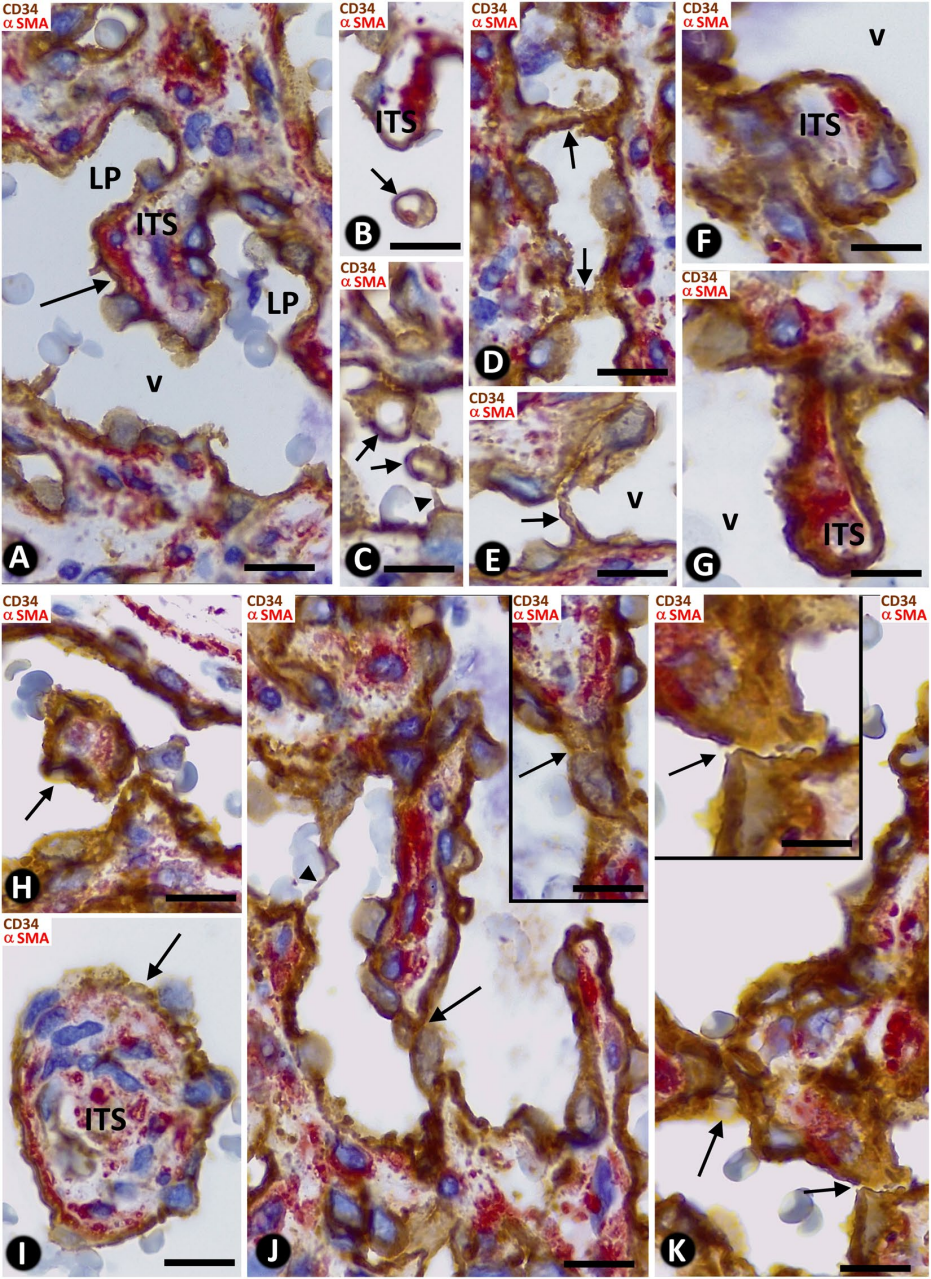
Vessel loops with scarce interendothelial contacts, open lumens, and intravascular pillars. Pillars (arrows) are covered by CD34^+^ ECs (corresponding to the inner layer of the loop) and show a core whose connective content (collagen, pericytes and interstitial cells) depends on pillar size. The loop open lumen (LP) is connected to that of the mother vessel (v) and surrounds an ITS (**A,B**). Nascent pillars are also observed (C and J, arrowhead). Note that pillars/ITSs are isolated in the vessel lumen (H and I, arrows) or adhered to the vessel wall, with a planar (**F**) or meso-like morphology (**G**), or to other pillars/ITSs, arranged linearly or grouped irregularly (**J**,**K**). The zones of adherence are shown at higher magnification in inserts of (**J**,**K**) (arrows). Double-staining with anti-CD34 (brown) and αSMA (red). Bar: (**A**–**K**) 8 µm.

**Figure 6:**
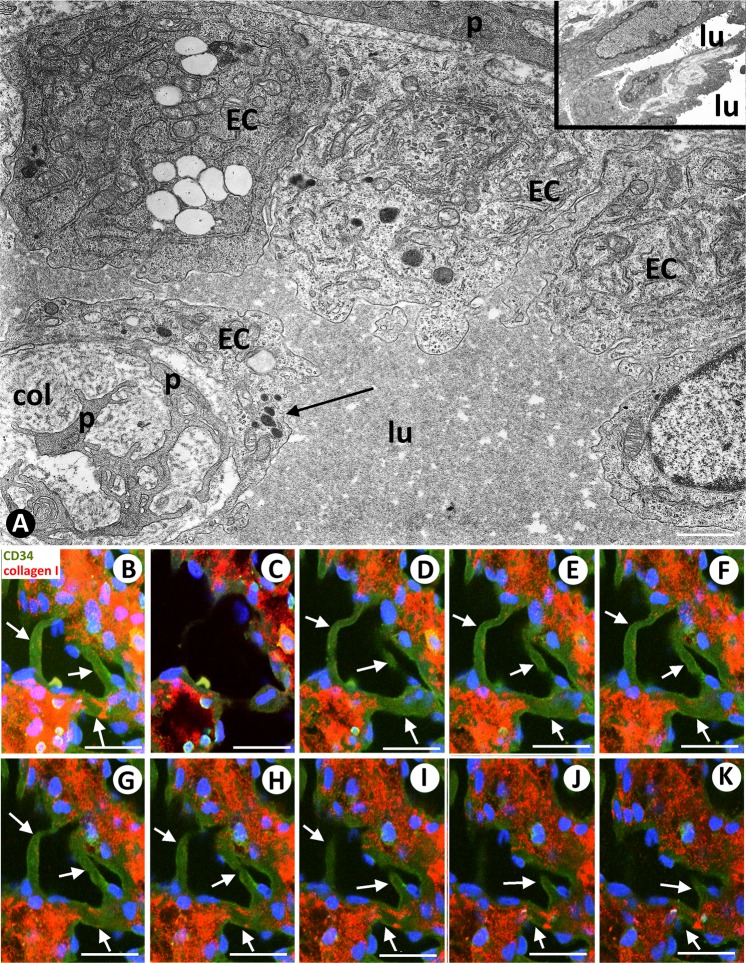
(**A**) Ultrastructural image of a zone of a vessel with prominent ECs and a transversally sectioned intravascular pillar (arrow), showing a cover formed by ECs and a core with processes of pericytes (p) and collagen material (col). In the insert, a pillar appears longitudinally sectioned. lu: vessel lumen. (**B**–**K**) The appearance and disappearance and the whole-mounted view (**B**) of three intravascular pillars (arrows) are shown in confocal microscopy. **A** and insert: Ultrathin sections. Uranyl acetate and lead citrate. (**B**–**K**) confocal microscopy, frontal view, 10 µm section, immunofluorescent label with anti-CD34 (green), anti-collagen I (red), DAPI (blue). Bar: (**A**) 2 µm; (**B**–**K**) 20 µm.

**Figure 7:**
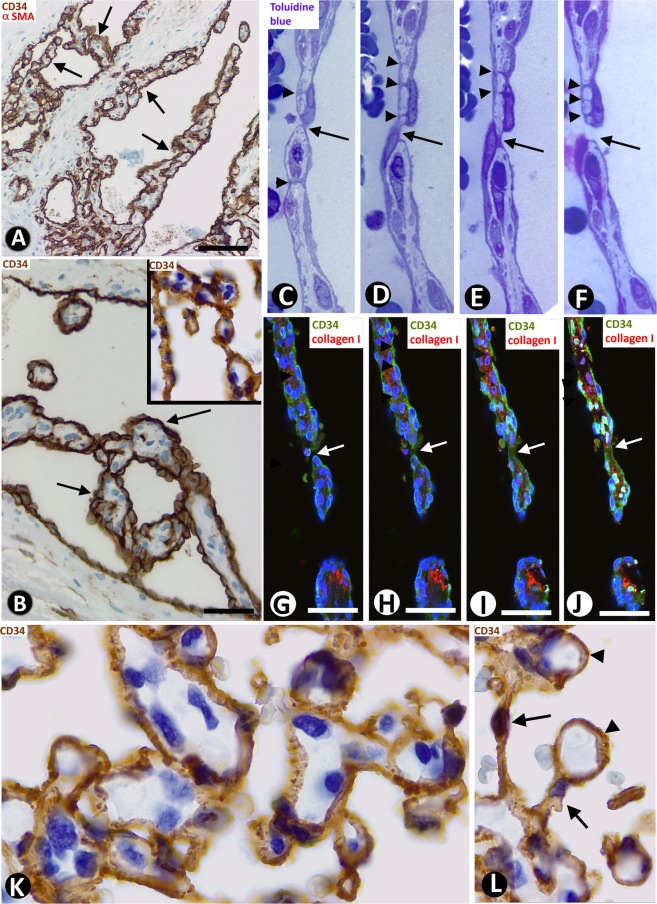
(**A**–**J**) Zones with sinusoidal hemangioma-like morphology are shown in LCH. (**A**–**B**) Pillars arranged in a linear fashion are observed forming incomplete septa (arrows), covered by CD34^+^ ECs. (**C**–**F**) In serial semithin sections, communications between opposite endothelial cells of the septa (arrowheads) and a zone of continuity and discontinuity between pillars are present (arrows). (**G**–**J**) Similar aspect of septa showing continuity and discontinuity between pillars (arrows) in confocal microscopy [single (**G**–**I**) and whole-mount (**J**) in frontal view (6 µm section)]. (**K**,**L**) Zones with IPEH-like morphology. Note the irregular arrangement of pillars (**K**), and large pillars (**L**-arrowheads) connected by thin pillars (**L**-arrows). (**A**) Section double-stained with anti-CD34 (brown) and anti-αSMA (red). (**B**,**K**,**L**) Sections stained with anti-CD34 (brown). (**C**–**F**) Serial semithin sections stained with Toluidine Blue. (**G**–**J**) Immunofluorescent label with CD34 (green), collagen I (red) and DAPI (blue). Bar: (**A**) 80 µm; (**B**–**J**) 20 µm; (**K**,**L**) 10 µm.

We also observed structures typical for IA, the main focus of our study, including pillar-associated structures and pillars/folds. The pillar-associated structures comprised vessel loops (Fig. 2H–K and 3A,D), contacts between ECs from opposite walls in the loops (including peg-like structures) (Fig. 3E–Z), perforated interendothelial contacts (Fig. 4A–G) and loops fragmented into capillary-sized vessels (capillary-like structures) (Fig. 4H–J). Pillar/folds were observed in varying number, size and arrangement (Figs. 5–7 and Table 1). These findings will be described below.

### Vessel loops in LCH

Numerous elementary and complex vessel loops, originating from venules or from other loops, were observed in histological sections (Figs. 2H–K and 3A–D). The loops were lined by ECs, which also expressed CD34 (Fig. 2H). Anti-αSMA^+^ pericytes and/or their processes could be present around the ECs (Fig. 2H). SA phenomena were observed in the mother vessels and in the growing loops (see above). Serial semithin sections, and single and whole-mounted images in confocal microscopy revealed that the double layer of the loop ECs was formed by endothelial sheets (Figs. 2I–K and 3A–D). Each loop, which had two segments connecting to the mother vessel (Fig. 2I–K), dissected and encircled an ITS (Fig. 2H–K).

### Contacts between ECs from opposite walls of the loop

Contacts were observed between ECs from opposite vessel walls of the loops (Fig. 3E–Z). These contacts varied depending on the morphology of the contacting cells, contact extension, symmetry or asymmetry, and distribution. In histologic sections, the contacting ECs could have a triangular, ovoid or flattened morphology (Fig. 3E–G). Contact surface extension was related to EC morphology: generally small when ECs where triangular (small apical contact) (Fig. 3E) and extensive when ECs were flattened (extensive planar contact) (Fig. 3G,H). In the symmetric contact (kissing contact), ECs protruded from both opposite walls of the vessels (Fig. 3E,F), whereas in the asymmetric contact, ECs only protruded from one side of the vessel wall (Fig. 3I,J). Depending on their distribution, the contacts could appear relatively grouped (multiple contacts) (Fig. 3K) or isolated (single contact) (Fig. 3I). ECs showing a triangular morphology in multiple symmetric contacts acquired a double serrated image. Contacts could also be formed by ECs or by EC protrusions that surrounded connective tissue, acquiring a peg-like image (Fig. 3L). Occasionally, extremely fine transcapillary EC projections adopting a filiform or antenna-like morphology were observed (Fig. 3M). In electron microscopy, some of these structures appeared as endothelial projections separating luminal spaces (Fig. 3N). Serial semithin sections showed the appearance and disappearance of these contacting cells (Fig. 3O–R). Likewise, in confocal microscopy, we also observed the appearance and disappearance in series of individual views (Fig. 3T–Z) and in whole-mounted view (Fig. 3S) of some of the aforementioned EC contacts.

### Frequency, distribution and perforation of interendothelial contacts in vessel loops

Most vessel loops showed numerous interendothelial contacts and the loop lumen was alternately open and virtual (Fig. 3K). In a smaller number of loops, the interendothelial contacts were absent or scarce, and the loop lumen was open (see below).

Perforation of endothelial contacts was seen in serial semi-thin sections (Figs. 4A,B). In these perforations (Fig. 4B), the core was formed by cytoplasmic extensions of pericytes, interstitial and inflammatory cells, and by collagen fibrils. The appearance and disappearance of these perforated interendothelial contacts were demonstrated in sequential images in confocal microscopy (Fig. 4C–G).

### Loop fragmentation in capillary-sized vessels with numerous interendothelial contacts

In the vessel loops with numerous interendothelial contacts alternating with capillary-like open lumen, when the contacts were perforated, the loop zones with open lumen appeared as isolated capillary-sized vessels or capillary-like spaces, arranged in a linear arciform path reminiscent of that of the loop (Fig. 4H–J). In the capillary-sized vessels, blood red cells were seen (Fig. 2I–K). The ITSs encircled by the loops appeared extravascular (Figs. 2H–K; 4H–J).

### Intravascular pillars in vessel loops with scarce or no interendothelial contacts. Zones with sinusoidal hemangioma-like and IPEH-like morphology

In vessel loops with scarce or no interendothelial contacts and wide lumen, small (≤2.5 µm) and large (>2.5 µm) pillars were formed in the vessel lumen (Fig. 5). Intravascular pillars showed a cover and a core, and were irregularly distributed. The cover was formed by ECs expressing CD34 (Fig. 5). These ECs corresponded to the loop inner layer. The content of the core varied depending on pillar size. The smaller of these structures presented a core that contained collagen material and cell extensions of pericytes and fibroblasts. The larger showed connective tissue components (Fig. 5), occasionally with small capillaries in giant pillars. Nascent pillars formed by extensions of ECs were also seen (Fig. 5C). Ultrastructurally, pillars characteristics were confirmed, observing covering ECs and processes of pericytes and collagen in the pillar core (Fig. 6A and insert). In confocal microscopy, we observed the appearance and disappearance in series of individual views of neighboring pillars (Fig. 6C–K), as well as their image in whole-mounted view (Fig. 6B).

Pillars could appear isolated in the vessel lumen (Figs. 5H,I) or adhered to other structures (Fig. 5F,G), such as the vessel wall and other pillars (Fig. 5J,K). When the intravascular pillars appeared joined together, they adopted a linear arrangement, forming incomplete septa (Fig. 7A–J), or were grouped irregularly (Fig. 7K,L). The septa frequently had a moniliform surface and were covered by CD34^+^ ECs (Fig. 7A,B) and the core presented αSMA^+^ cells with a pericyte-like aspect, together with other stromal cells. Communications between opposite endothelial cells within each septum and zones of continuity and discontinuity between the pillars that formed each septum were observed in serial semithin sections and in confocal microscopy (Fig. 7C–J). The zones presented above with a rise in these phenomena showed a sinusoidal hemangioma-like morphology (presence of incomplete septa by linear arrangement of pillars) (Figs. 1C, 7A,B) or IPEH-like morphology (numerous pillars grouped irregularly) (Figs. 1D and 7K,L). In IPEH, large pillars connected by thin pillars were frequently observed (Fig. 7L).

### Relation between vessel loops and venules

The elementary loops formed from the wall of the branched intralobular vein originated from the venule girth or from one side of the venule. From these elementary loops, new loops developed and formed the complex lobular architecture characteristic of this lesion.

## Discussion

In this work, we report the participation of IA in synergistic interaction with SA in LCH and confirm several morphogenic mechanisms involved in the formation of pillars (hallmarks of intussusception) and capillary-like structures. In addition, we demonstrate that the prevalence of some intussusceptive mechanisms over others determines the morphologic patterns in LCH (predominant capillary pattern and focal sinusoidal hemangioma-like and IPEH-like patterns). Based on these findings and on previous results in sinusoidal hemangioma and in IPEH^14,16^, we suggest that intussusceptive mechanisms may participate in the morphogenesis of vascular tumours/pseudotumours in general. Finally, we take into account the possible histogenic implication of the venules in the lesion origin and lobular aspect.

The presence of pillars, folds and associated morphogenic structures, typical for IA, in LCH confirms that IA participates in LCH formation, a role previously only assigned to SA. The demonstration of these intussusceptive findings was undertaken in serial semithin sections, and in successive and whole-mounted images in confocal microscopy. The combination of SA and IA in LCH concurs with that observed by different authors in the chick chorioallantoic membrane, and in developmental avian kidney and lung^2,13,18,19^. This synergistic action between SA and IA has also been observed in the rat femoral vein after PGE2 and glycerol perivenous administration and in the zebrafish caudal vein plexus^15,17^. By these complementary mechanisms, in an initial phase, vessels mainly grow by sprouting, followed by intussusception. The presence of blood red cells in the lumen of the capillary-sized spaces formed in the loops suggests that the loops are generally perfused and kissing or peg-like contacts are established. Secondary structures formed by fusion or splitting of pillars can also occur, originating intravascular meshworks of processes, septa or irregular aggregates (see below).

The intussusceptive precursor findings involved in pillar morphogenesis in LCH coincide with those previously proposed by pioneering authors studying intussusception^3,6,10–13,20^ and their frequency in LCH indicates that this lesion is an appropriate substrate for the study of these morphogenic mechanisms. Thus, the findings in this work support pillar formation by kissing endothelial and peg-like contacts, meso-like intraluminal folds, split intercapillary meshes and vessel loops encircling ITSs.

**Figure 8:**
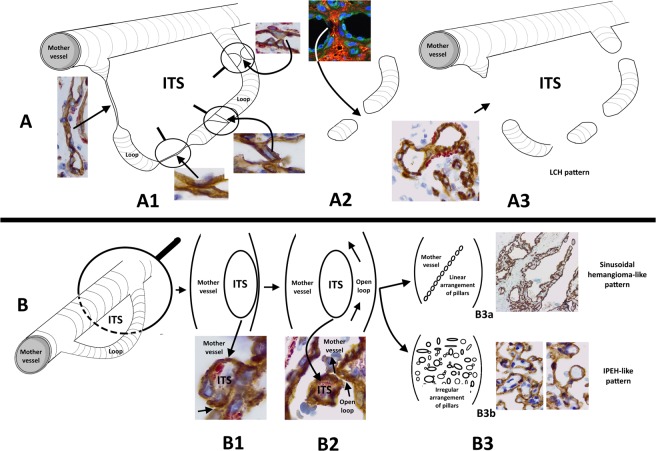
Schematic representation of the IA mechanisms that influence vascular lesion morphogenesis, depending on the formation, or not, of multiple interendothelial contacts. (**A**) Successive steps of capillary-like space formation are shown in A1, A2 and A3, with their corresponding microphotographs. In A1, a loop originating from a mother vessel. The loop shows several examples of interendothelial contacts, alternating with capillary-like spaces. In A2, perforation of an interendothelial contact. In A3, loop segmented in capillary-like structures, acquiring the capillary aspect of LCH. These images only represent one loop. Spatially, however, multiple converging loops occur in the lesion, and the capillary-sized spaces may connect with others in other loops. These findings could explain the future conservation or involution of these spaces. Note that the ITS surrounded by the loop remains outside the mother vessel (extravascular). (**B**) Several steps of pillar formation in the mother vessel are shown in B1, B2 and B3, with their corresponding microphotographs. The loop originating from the mother vessel (B1) becomes permeable (B2), and the ITS and its surrounding endothelium (internal side of the loop) is transported to the mother vessel lumen (although some connections between the pillar and vessel wall may persist). In B3, several pillars can be arranged linearly or irregularly, originating the sinusoidal-hemangioma-like (B3a) or IPEH-like (B3b) pattern, respectively.

Loops encircling ITSs and interendothelial contacts between the opposite walls of the loops were common findings in LCH. The frequency of interendothelial contacts between the opposite walls of the loops conditions the LCH patterns (see scheme in Fig. 8 and Table 1). When numerous interendothelial contacts occur in the loops, and the contacts are perforated, the intercalated open lumens of the loops between the contacts persist as capillary-sized vessels (main capillary pattern of the lesion) arranged according to the primitive loop path. When few inter-endothelial contacts occur, the loop lumen is patent, and the inner side of the loop forms the cover of intraluminal pillars (the core corresponds to the ITS encircled by the loop), which may be arranged linearly, forming septa (focal sinusoidal-like pattern) or may be grouped in an irregular form (focal IPEH-like pattern). These intussusceptive mechanisms can be linked to hemodynamic conditions in the loops and mother vessels, as occurs in those described in vascular expansion and branching remodelling^1,6,21–26^, including the influence of intraluminal flow fields^1,23,25,26^.

This work and previous studies^14,16^ show that LCH, sinusoidal hemangioma and IPEH present different morphologic patterns and share hallmarks of intussusception, in which vessel loops have an important morphogenic role. A similar participation of vessel loops has been demonstrated in the ovarian vein of the rat after ovariectomy and human tumour xenograft implantation^10,11^ and in other human blood and lymphatic vessel diseases, such as hemorrhoidal disease, lymphangiomas/lymphatic malformations and vascular transformation of lymph node sinuses, as well as in some sinuses of developing human foetal lymph nodes^5,14,27–29^. We use the term “piecemeal form of intussusceptive angiogenesis” to describe the mechanism of the formation and intravascular transport of pillars by vessel loops^5,14–16,30^. The morphologic differences of sinusoidal hemangioma, IPEH and LCH depend on the number of interendothelial contacts between the opposite walls of the loops and intravascular pillars formed in/from vessel loops, as well as on the substrate in which vessel loops and pillars form and on the different arrangement of pillars (see Fig. 8 and Table 1). Therefore, the pattern of these lesions depends on the predominance of certain intussusceptive mechanisms. Further studies are required to assess whether intussusception participates in other vessel tumours/pseudotumours and whether the prevalence of certain intussusceptive mechanisms also conditions their morphologic pattern.

Importantly, LCH is a process that can show spontaneous regression with relative frequency. Intussusceptive branching remodelling is a mechanism described in vessel bifurcations, with regression, retraction and atrophy of superfluous vessels^6–8^. The transcapillary pillars in LCH could somehow achieve the same effect as the formation of transvascular pillars in vessel bifurcations (now occurring as multiple pillars in smaller vessels), which would explain LCH regression. This issue also requires further study.

The observation of a variably branched venule giving rise to numerous microvessels in each LCH lobule suggests that the lobular pattern of the lesion depends on its multi-venular origin. Namely, each primitive venule originates one lobule, which is separated from other lobules by primitive inter-venular connective tissue. The central or eccentric location of the venule in the lobule can depend on whether capillaries originate from the entire venule girth or from one side, a process that can be observed in initial phases of lobule formation. Subsequent intense capillary formation from other neo-vessels increases the complexity of lobule architecture. In some lobules, the branched venule and new vessels with virtual lumens form a highly cellular structure with intercalated venular and capillary lumens.

## Material and Methods

### Human tissue samples

The archives of Histology and Anatomical Pathology of the Departments of Basic Medical Sciences of La Laguna University, University Hospital, and Eurofins® Megalab–Hospiten Hospitals of the Canary Islands were searched for cases of LCH for the period 1990–2017. Paraffin blocks were obtained from surgical specimens of 120 Caucasian patients: 56 males (46.66%) and 64 females (53.33%), ages ranging from 5 to 75 years, predominantly in the second and third decades of life (48 cases). Lesion evolution ranged from 12 days to 3 years. The samples of the 120 cases were studied by conventional histologic techniques. From them, 20 cases with more evident zones of sinusoidal hemangioma-like (n: 11) and IPEH-like (n: 9) patterns were used for immunochemistry procedures and immunofluorescence in confocal microscopy. Likewise, 10 cases were obtained for serial semithin and ultrathin sections. Most of the specimens selected corresponded to the earliest lesions (n: 18). Ethical approval for this study was obtained from the Ethics Committee of La Laguna University (Comité de Ética de la Investigación y de Bienestar Animal, CEIBA2019-0339), including the dissociation of the samples from any information that could identify the patient. The authors therefore had no access to identifiable patient information.

### Light microscopy

Specimens for conventional light microscopy were fixed in a buffered neutral 4% formaldehyde solution, embedded in paraffin and cut into 3 μm-thick sections. Sections were stained with Haematoxylin and Eosin (H&E), Trichrome staining (Roche, Basel, Switzerland. Ref. 6521908001) and Reticulin staining (Roche, Ref. 05279399001).

Specimens for semithin sections (1 µm) were fixed in a glutaraldehyde solution, diluted to 2% with sodium cacodylate buffer, pH 7.4, for 6 hours at 4 °C, washed in the same buffer, post-fixed for 2 hours in 1% osmium tetroxide, dehydrated in a graded ethanol series, and embedded in epoxy resin. Serial semithin sections were mounted on acid-cleaned slides, stained with 1% Toluidine blue (Merck®), and observed under a Leica® DM-750 light microscope with an integrated High Definition Camera.

### Immunohistochemistry

Histologic sections, 3 μm-thick, were attached to silanized slides. After pre-treatment for enhancement of labelling, the sections were blocked with 3% hydrogen peroxide and then incubated with primary antibodies (10–40 minutes). The primary antibodies (Dako, Glostrup, Denmark) used in this study were CD34 monoclonal mouse anti-human, clone QBEnd-10, (dilution 1:50), catalog No. IR632 and α-smooth muscle actin (αSMA) monoclonal mouse anti-human, clone 1A4 (dilution 1:50), catalog No. IR611. The immunoreaction was developed in a solution of diaminobenzidine and the sections were then briefly counterstained with haematoxylin, dehydrated in ethanol series, cleared in xylene and mounted in Eukitt®. Positive and negative controls were used. For the double immunostaining, we used anti-CD34 antibody (diaminobenzidine, DAB, as chromogen) to highlight CD34^+^ ECs and anti-αSMA (aminoethylcarbazole, AEC, substrate-chromogen) for anti-αSMA^+^ pericytes/smooth muscle cells.

### Immunofluorescence in confocal microscopy

For immunofluorescence, tissue sections of 6 and 10 µm were obtained. For antigen retrieval, sections were deparaffinized and boiled for 20 minutes in sodium citrate buffer 10 mM (pH 6), rinsed in Trisbuffered saline (TBS, pH 7.6, 0.05 M), and incubated with the following primary antibodies diluted in TBS overnight in a humid chamber at room temperature: mouse monoclonal anti-CD34, code no. IR63261 (ready to use), rabbit polyclonal anti-collagen type I (1/100 dilution, code AB749P, Millipore). For the double immunofluorescence staining, sections were incubated with a mixture of monoclonal and polyclonal primary antibodies (mouse monoclonal anti-CD34 and rabbit polyclonal anti-collagen type I). The next day, the slides were rinsed in TBS and incubated for 1 h at room temperature in the dark with the secondary biotinylated goat anti-rabbit IgG (H + L) (1:500, Code: 65-6140, Invitrogen, San Diego, CA, USA) and Alexa Fluor 488 goat antimouse IgG (H + L) antibody (1:500, Code: A11001, Invitrogen), followed by incubation with Streptavidin Cy3 conjugate (1:500, Code: SA1010, Invitrogen) for 1 h at room temperature in the dark. Nuclei were detected by DAPI staining (Chemicon International, Temecula, CA, USA). After washing in TBS, sections were exposed to a saturated solution of Sudan black B (Merck, Barcelona, Spain) for 20 minutes to block autofluorescence. They were rinsed in TBS and cover-slipped with DABCO (1%) and glycerol-PBS (1:1). Negative controls were performed in the absence of primary antibodies. Fluorescence immunosignals were obtained in a Fluoview 1000 laser scanning confocal imaging system (Olympus Optical) using the objective 60×/1.35 oil.

### Ethics approval

Ethical approval for this study was obtained from the Ethics Committee of La Laguna University (CEIBA2019-0339), including the dissociation of the samples from any information that could identify the patient. The authors therefore had no access to identifiable patient information.

## Data Availability

Availability of materials (e.g. reference of antibodies) and data are included in this work. We confirm that all methods were performed in accordance with the relevant guidelines and regulations.
